# A Brazilian classified data set for prognosis of tuberculosis, between January 2001 and April 2020

**DOI:** 10.1038/s41597-022-01892-4

**Published:** 2022-12-15

**Authors:** Maicon Herverton Lino Ferreira da Silva Barros, Guto Leoni Santos, Maria Gabriela de Almeida Rodrigues, Vanderson Sampaio, Theo Lynn, Patricia Takako Endo

**Affiliations:** 1 Universidade de Pernambuco (UPE), Programa de Pós-graduação em Engenharia de Computação (PPGEC), Recife, 50720-001 Brazil; 2 Universidade Federal de Pernambuco (UFPE), Centro de Informática (CIn), Recife, 50740-560 Brazil; 3 Universidade Estadual do Amazonas (UEA), Programa de Pós-graduação em Medicina Tropical (PPGMT), Manaus, 69850-000 Brazil; 4 Instituto Todos pela Saúde (ITpS), São Paulo, 01310-942 Brazil; 5 Dublin City University (DCU), Dublin, Ireland

**Keywords:** Disease-free survival, Public health, Tuberculosis

## Abstract

After COVID-19, tuberculosis (TB) is the leading cause of death by an infectious disease in the world. This work presents a data set based on data collected from the Brazilian Information System for Notifiable Diseases (SINAN) for the period from January 2001 to April 2020 relating to patients diagnosed with tuberculosis in Brazil. The data from SINAN was pre-processed to generate a new data set with two distinct treatment outcome classes: CURED and DIED. The data set comprises 37 categorical attributes (including socio-demographic, clinical, and laboratory data) as well as the target class. There are 927,909 records of patients classified as CURED and 36,190 classified as DIED, totaling 964,099 records.

## Background & Summary

Tuberculosis is an airborne infectious disease caused by the bacillus *Mycobacterium tuberculosis*; globally it is the second largest cause of morbidity and mortality by an infectious agent^1,2^. Historically, there has been a significant global effort to reduce the death rate of tuberculosis. However, these efforts have been compromised due to the COVID-19 pandemic. Brazil has one of the highest incidences of tuberculosis worldwide and is among the 22 countries considered by World Health Organization (WHO) as having a high burden of tuberculosis^3,4^. In 2019, Brazil registered 96,000 cases of the disease, with a mortality rate of 7.00%0^4^.

The elimination of tuberculosis is a global priority, as evidenced by its inclusion in the Sustainable Development Goals. Central to reducing the transmission of TB and ultimately the elimination of TB is early identification of TB-infected patients, application of infection-control measures, and early enrollment in treatment^5^. To this end, WHO has called for intensified research and innovation to improve early diagnosis, shorten and provide more effective treatment regimens, improve prevention, and partners for cross-sectoral actions^5^.

The clinical management of tuberculosis relies on the medical assessment of clinical and diagnostic information. Data on relapse, co-infection, and severity can be crucial to decide on procedures as pharmacological and clinical interventions. Timely intervention is vital to control the spread of the disease, and the patient’s prognostis and ultimate outcome. However, predicting a patient’s prognosis is a complex task as tuberculosis has different treatment outcomes depending on the type of TB^6^. Answering the WHO call for innovation in early diagnosis, extant literature has proposed the application of artificial intelligence techniques, such as machine learning and deep learning models, to support the speed and efficacy of tuberculosis treatment decision-making, and specifically prognosis.

The Brazilian Information System for Notifiable Diseases (Sistema de Informação de Agravo de Notificação or SINAN) from the Brazilian Ministry of Health collects and stores data on each disease incidence of a notifiable disease in Brazil. This data is routinely generated by the Epidemiological Surveillance System. SINAN has a database with socio-demographic, clinical, and laboratory data on suspected tuberculosis cases that can be used to generate multiple analyses for public health planning and the assessment of disease prognosis. However, most machine learning and deep learning models applied in the literature for the treatment of tuberculosis require labeled data, that is, they contain information about what is being classified. This work presents an extension of the SINAN database that includes outcome data (i.e. “CURED” or “DIED”) for the period January 2001 to April 2020. The availability of such data enables researchers to create training and test data sets, and use this data to build, evaluate, and optimise machine learning models to support the prognosis of tuberculosis in patients. Also, other outcomes regarding treatment adherence and relapses are available and can be assessed. A high-level epidemiological analysis of the data set is also presented.

## Methods

The original data was collected from the Information System for Notifiable Diseases (*Sistema de Informação de Agravos de Notificação*^7^) for the period from January 2001 to April 2020 including data from all 26 Brazilian states and the Federal District (Brasília) of Brazil. It contains socio-demographic, clinical and laboratory data about patients who were diagnosed with tuberculosis. While the SINAN-TB database is public, certain data is labeled sensitive and is protected by the General Law for the Protection of Personal Data Brazil (*Lei Geral de Prote*çã*o de Dados Pessoais* or LGPD). Such sensitive data is only available upon request to SINAN’s ethics committee. The data used in this research does not contain any such sensitive information.

**Fig. 1:**
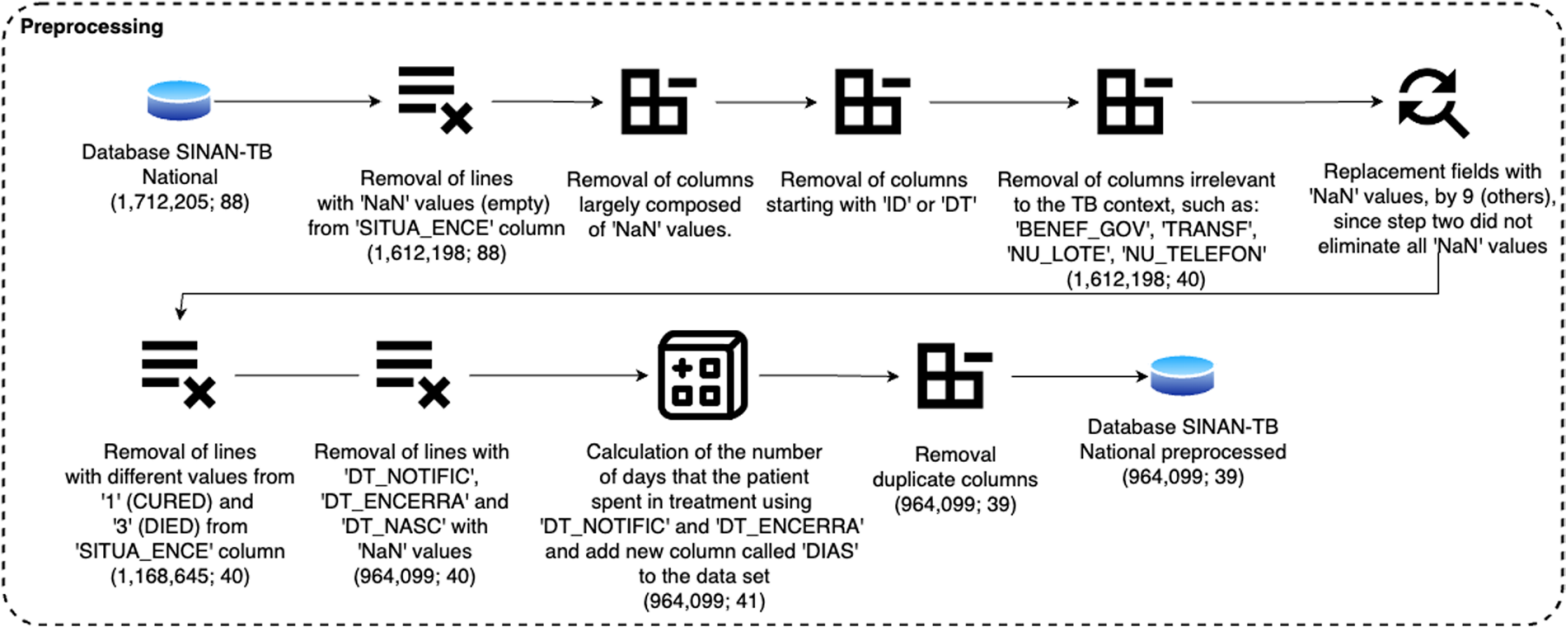
Pre-processing steps performed to build the final data set.

The SINAN data set was cleaned using a variety of preprocessing techniques as outlined in Fig. 1. The original data set comprised 1,712,205 records and 88 attributes. Following preprocessing, 748,106 rows and 50 fields were removed resulting in a final preprocessed data set of 964,099 records and 38 attributes.

Tables 1–4 shows all the attributes removed in the preprocessing process. These attributes were removed for different reasons including the column featuring primarily empty values (‘NaN’); attributes starting with the nomenclature ‘ID’; attributes starting with ‘DT’ with the exception of ‘DT_NOTIFIC’ and ‘DT_NASC’; attributes irrelevant to the tuberculosis context (such as ‘BENEF_GOV’, ‘TRANSF’, ‘NU_LOTE’ and ‘NU_TELEFON’); replacement fields with ‘NaN’ values, by 9 (others), since step two did not eliminate all ‘NaN’ values; removal of lines with different values from ‘1’ (CURED class) and ‘3’ (DIED class) from the attribute ‘SITUA_ENCE’; removal of lines with ‘DT_NOTIFIC’, ‘DT_ENCERRA’ and ‘DT_NASC’ with ‘NaN’ values; calculation of the number of days that the patient spent in treatment using ‘DT_NOTIFIC’ and ‘DT_ENCERRA’ and add new attribute called ‘DIAS_EM_TRATAMENTO’; attributes removed by authors’ discretion/analysis, as well as duplicate data and attributes.

**Table 1:** Attributes removed from original SINAN-TB database - Reason for removal: more than 65.00% of records are null.

Attribute	Description
BENEF_GOV	Reports whether the patient receives government benefits
DT_MUDANCA	Treatment change date
TRANSF	Informs if the patient was transferred.
UF_TRANSF	Federative unit from which the patient was transferred
AGRAVOUTDE	Inform if other associated grievances specify
ANT_RETRO	Antiretroviral treatment
BAC_APOS_6	Result of sputum smear microscopy for Acid-fast bacillus (AFB) performed on a sample collected after the 6st month of treatment
EXTRAPU1_N	Extrapulmonary location of tuberculosis
EXTRAPU2_N	Extrapulmonary location of tuberculosis
EXTRAPUL_O	Others extrapulmonary location of tuberculosis
ID_OCUPA_N	Sequential identifier that refers to another table in the database referring to the profession exercised by the patient.
OUTRAS_DES	Inform if other types of drugs (Specify)
POP_IMIG	Informs if the patient is an immigrant
POP_LIBER	Informs if the patient is incarcerated
POP_RUA	Informs if the patient is homeless
POP_SAUDE	Informs if the patient is undergoing any treatment
SITUA_12_M	Result of treatment with a 12-month regimen.
TEST_MOLEC	Informs if the molecular test was performed
TEST_SENSI	Informs whether the sensitivity test
MIGRADO_W	Identifies if the record comes from the Windows base migration routine
MUN_TRANSF	Municipale unit from which the patient was transferred

**Table 2:** Attributes removed from original SINAN-TB database - Reason for removal: outside the socio-demographic, clinical and/or laboratory context.

Attribute	Description
ID_AGRAVO	Sequential identifier that refers to another table in the database referring to the disease. In which case, all records refer to patients diagnosed with tuberculosis.
ID_MN_RESI	Sequential identifier that refers to another table in the database referring to the municipality where the patient resides.
ID_MUNIC_2	Sequential identifier that refers to another database table referring to the municipality where the patient was registered in the system.
ID_MUNIC_A	Sequential identifier that refers to another table in the database referring to the municipality responsible for monitoring the patient.
ID_MUNICIP	Sequential identifier that refers to another database table referring to the municipality where the patient was registered in the system.
ID_PAIS	Sequential identifier that references another database table referring to the patient’s country.
ID_REGIONA	Sequential identifier that refers to another table in the database referring to the patient’s region.
ID_RG_RESI	Sequential identifier that references another table in the database
IN_VINCULA	Sequential identifier that references another table in the database referring to notification investigation data.
NDUPLIC_N	Duplication of system categories
NU_ANO	Duplicate notification year with ‘DT_NOTIFIC’ column
NU_COMU_EX	Indicate the number of contacts examined in the investigation of the notified case
NU_CONTATO	Indicate the Number of Contacts informed at the time of diagnosis of the case
SG_UF	Acronym of the Federated Unit of residence of the patient at the time of notification
SG_UF_2	Acronym of the Federated Unit of residence of the patient at the time of notification
SG_UF_AT	Acronym of the Federated Unit of residence of the patient at the time of notification
Unnamed: 0	Index generated by the pandas dataframe library (Python).

**Table 3:** Attributes removed from original SINAN-TB database - Reason for removal: removed by authors’ discretion/analysis.

Attribute	Description
CS_ESCOL_N	Patient’s education
CS_GESTANT	Patient’s gestational age.
CULTURA_OU	Result of culture of other material for M. tuberculosis performed on a sample for diagnosis
DT_DIAG	Date of diagnosis
DT_INIC_TR	Treatment start date
DT_NOTI_AT	Date of case notification
HISTOPATOL	Result of histopathological examination for diagnosis of TB
INSTITUCIO	Institutional situation of the patient such as being in prison, in an asylum and others.
SITUA_9_M	Result of treatment with a 6-month regimen.
TP_NOT	Identifies the type of notification
TPUNINOT	Sequential identifier that refers to another table in the database referring to the type of notification.
TRATSUP_AT	Inform whether supervised treatment was performed until the closure of the Case

**Table 4:** Attributes removed from original SINAN-TB database - Reason for removal: Removed for other reasons.

Attribute	Description
DT_ENCERRA	End date of treatment outcome
SG_UF_NOT	Federative Unit where the health unit (or other notifying source) that made the notification is located
NU_IDADE_N	Patient age

## Data Records

The original and preprocessed data set, as well as the English data dictionary, are available at the Mendeley Data repository and can be accessed via the link (10.17632/fkpfd5b9n9.5)^8^.

**Fig. 2:**
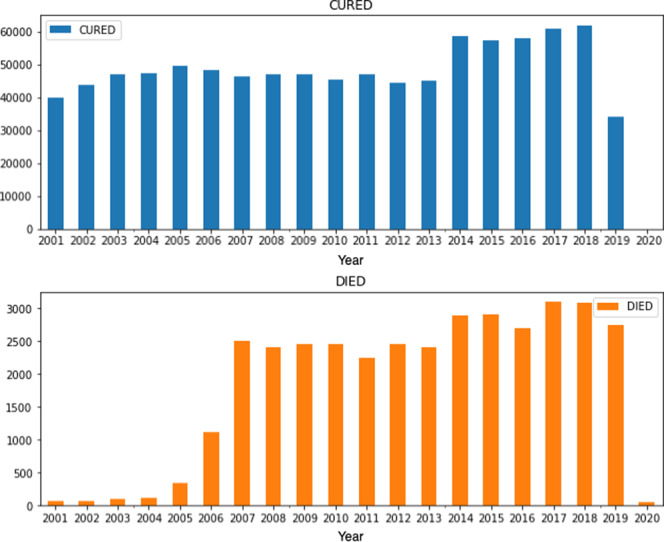
Records in the data set by year and by prognosis (records labelled as CURED and DIED).

Figure 2 presents the number of records in the data set by year and by prognosis (records labelled as CURED and DIED) in Brazil between January 2001 and April 2020. It is important to note that the year 2020 has relatively fewer records as the data set only includes records up to April 2020. In addition, SINAN notifications were adversely affected by the COVID-19 pandemic^2^. The highest number of DIED cases was in 2017 (3,099) and the highest number of CURED cases was in 2018 (61,839).

**Fig. 3:**
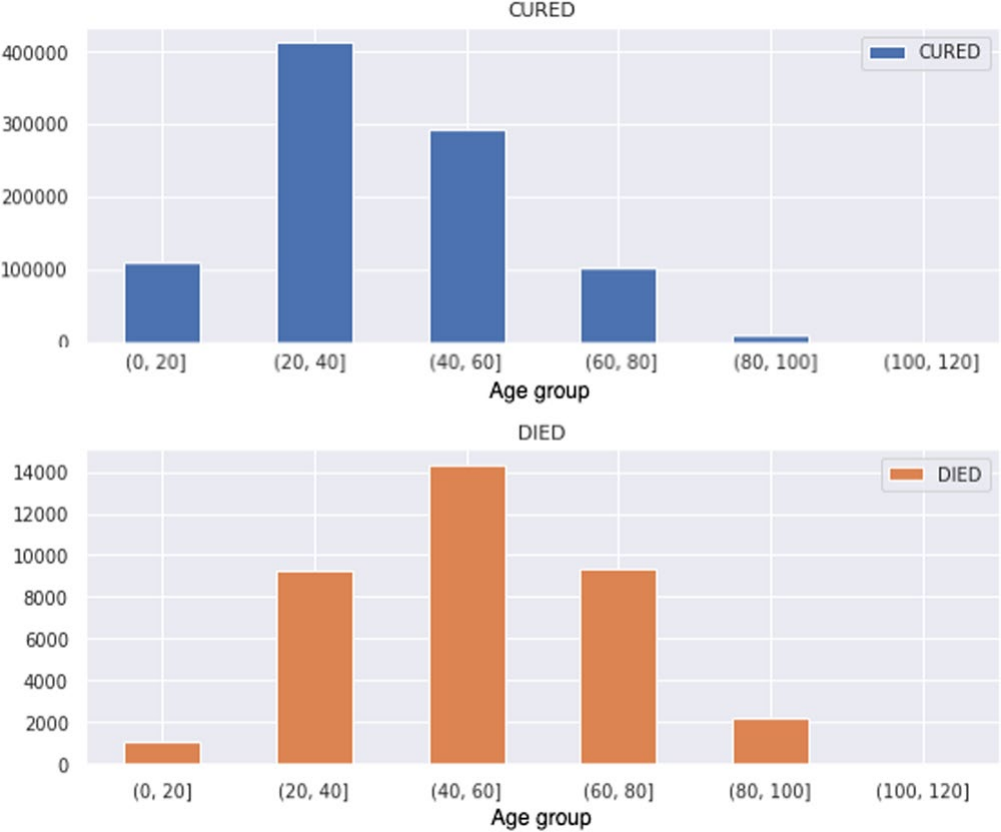
Records in the data set by age group and by treatment outcomes (records labelled as CURED and DIED).

Figure 3 presents the number of records in the data set by age group and by treatment outcome (records labelled as CURED and DIED). Most cases of tuberculosis are among patients 20 to 60 years old, with the highest number of CURED (412,723) in the 20 to 40 age group, and the highest number of DIED (14,349) between 40 and 60 years old.

**Fig. 4:**
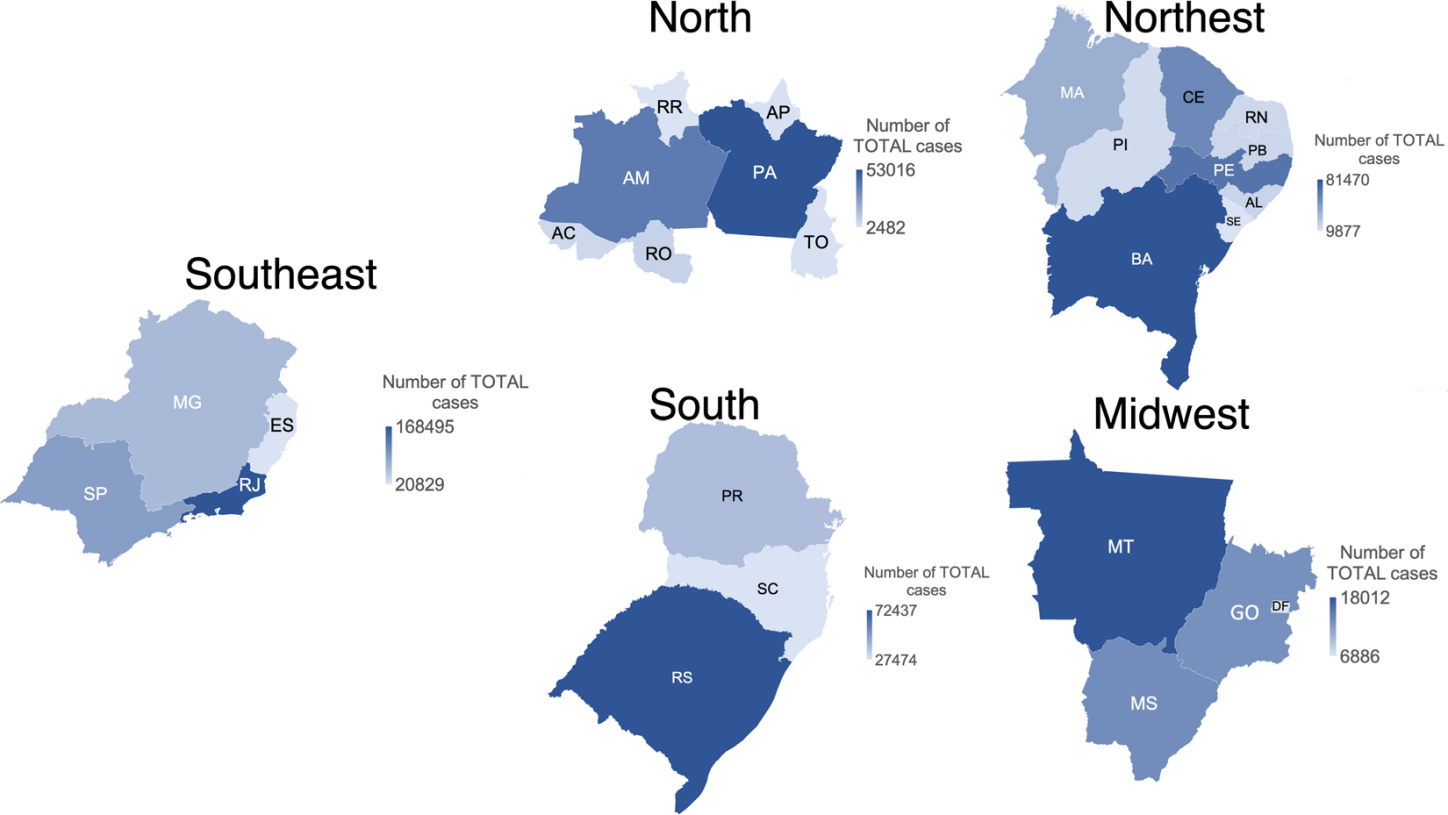
Confirmed cases of tuberculosis by Brazilian region between January 2001 and April 2020.

**Fig. 5:**
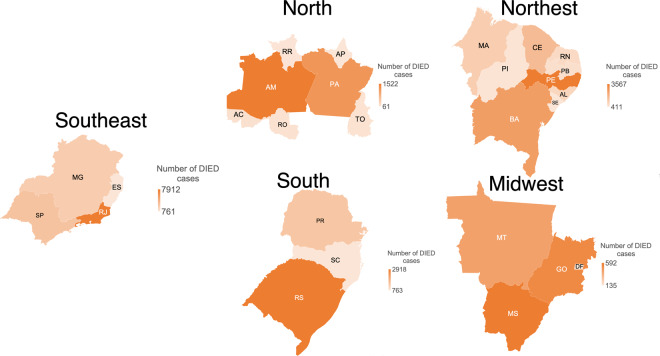
Deaths by tuberculosis by Brazilian region between January 2001 and April 2020.

Figure 4 presents heat maps of the cases of tuberculosis by Brazilian regions between January 2001 and April 2020, while Fig. 5 shows the cases of DIED by region in the same period. The Southeast region, comprising the states of São Paulo (SP), Minas Gerais (MG), Espírito Santo (ES), and Rio de Janeiro (RJ) had the highest incidence of tuberculosis with 345,491 cases (records labelled as CURED and DIED); it also had the highest number of deaths (14,215) over the 19 years. With 51,878 cases, the Midwest region was the region with the lowest number tuberculosis cases and lowest number of deaths (1,697). The state with the highest number of tuberculosis cases was Rio de Janeiro (RJ) with 168,495 tuberculosis cases and 7,912 deaths. The state with the lowest incidence of tuberculosis was Roraima (RR), in the North region, with 2,413 cases of TB. The state with the lowest incidence of deaths is Amapá (AP) with 61 registered deaths Table 5.

**Table 5:** Socio-demographic data.

Attribute	Description
DT_NOTIFIC	Date of notification of the case in the SINAN system.
CS_SEXO	Patient’s biological sex
CS_RACA	Race declared by the patient
SITUA_ENCE	Closing status of all reported cases
UF	State of the federal unit Brazil where the patient resides
DIAS_EM_TRATAMENTO	Number of days that the patient was in treatment calculated from the date of diagnosis to the date of the end of treatment
IDADE	Patient age

**Fig. 6:**
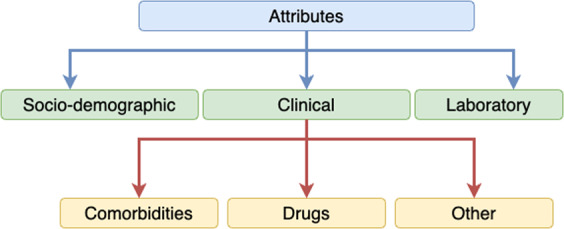
High level attribution categorisation in the final data set.

The final data set had 39 attribute grouped in to the three categories - socio-demographic (as presented in Table 5), clinical, and laboratory based on^9,10^. As can be seen in Fig. 6, clinical data was further categorised into comorbidities, drugs, and other.

Table 6 shows the attributes grouped as clinical data for comorbidities such as diabetes, AIDS and others. Drugs administered to patients during tuberculosis treatment were grouped as clinical data as per Table 7.

**Table 6:** Clinical data – Comorbidities.

Attribute	Description
AGRAVAIDS	AIDS associated with tuberculosis at the time of the notification
AGRAVALCOO	Alcohol consumption associated with tuberculosis at the time of the notification
AGRAVDIABE	Diabetes associated with tuberculosis at the time of the notification
AGRAVDOENC	Mental disease associated with tuberculosis at the time of the notification
AGRAVOUTRA	Other diseases associated with tuberculosis at the time of the notification
DOENCA_TRA	If the patient acquired the disease as a result of the working conditions/situation
AGRAVDROGA	Other drug consumption associated with tuberculosis at the time of the notification
AGRAVTABAC	Tobacco consumption associated with tuberculosis at the time of the notification

**Table 7:** Clinical data – Drugs.

Attribute	Description
RIFAMPICIN	Rifampicin drugs
ISONIAZIDA	Isoniazid drugs
ETAMBUTOL	Etambutol drugs
ESTREPTOMI	Streptomi drugs
PIRAZINAMI	Pyrazinami drugs
ETIONAMIDA	Ethionamide drugs
OUTRAS	Other drugs

Only two clinical attributes were labelled “Other” as per Table 8: the clinical form of tuberculosis (labelled as “FORMA”) and the type of health unit admission (labelled as “TRATAMENTO”) for the patient containing: new case, recurrence, re-entry after abandonment, don’t know, transfer and post-death.

**Table 8:** Clinical data – Other.

Attribute	Description
TRATAMENTO	Patient Health Unit Admission Type.
FORMA	The clinical form of tuberculosis at the time of notification by location location.

The laboratory attributes were generated from the results of tests performed in the laboratory such as X-ray, HIV serology result, tuberculin skin test etc, and were grouped as shown in Table 9.

**Table 9:** Laboratory data.

Attribute	Description
RAIOX_TORA	Result of chest X-ray at the time of notification (code 3 refers to other changes not compatible with tuberculosis)
TESTE_TUBE	Tuberculin skin test result: Non-reactor (0–4 mm), Weak reactor (5–9 mm), Strong reactor (10 mm or more)
BACILOSC_E	Result of sputum smear for Acid-Fast bacillus (AFB) performed on a sample for diagnosis - 1st sample
BACILOS_E2	Result of sputum smear for Acid-Fast bacillus (AFB) performed on a sample for diagnosis - 2nd sample
BACILOSC_O	Result of smear of other material for Acid-Fast bacillus (AFB) performed on a sample for diagnosis - 3rd sample
CULTURA_ES	Result of sputum culture for M. tuberculosis performed in sample for diagnosis
HIV	Result of serology for the acquired immunodeficiency virus, performed before or after the notification of TB. It aims to assess HIV co-infection
BACILOSC_1	Result of sputum smear microscopy for Acid-Fast bacillus (AFB) performed on a sample collected at the end of the 1st month of treatment
BACILOSC_2	Result of sputum smear microscopy for Acid-Fast bacillus (AFB) performed on a sample collected at the end of the 2nd month of treatment
BACILOSC_3	Result of sputum smear microscopy for Acid-Fast bacillus (AFB) performed on a sample collected at the end of the 3rd month of treatment
BACILOSC_4	Result of sputum smear microscopy for Acid-Fast bacillus (AFB) performed on a sample collected at the end of the 4th month of treatment
BACILOSC_5	Result of sputum smear microscopy for Acid-Fast bacillus (AFB) performed on a sample collected at the end of the 5th month of treatment
BACILOSC_6	Result of sputum smear microscopy for Acid-Fast bacillus (AFB) performed on a sample collected at the end of the 6th month of treatment

Supplementary Table 1 lists all attributes described with their appropriate characteristics. Males had the highest number of records labelled as CURED and DIED; females had a mortality rate almost three times lower than men (26.40%). Only 6.00% of tuberculosis cases had an AIDS-associated disease and 6.80% of patients tested positive for HIV. The most widely administered drugs were Rifampicin and Isoniazid, both with 67.00% of CURED cases, although 50.20% of patients who died from the disease also took these drugs. The drugs with a low administration rate were Streptomi and Ethionamide with only 0.80% and 0.90% of the total number of patients taking these medications, respectively. The pulmonary clinical form of tuberculosis represents 84.60% of all cases. Patients who died from tuberculosis spent an average of 56 days in treatment while those cured spent 211 days in treatment.

## Technical Validation

All data presented in this work can be corroborated by reports published by the Brazilian Ministry of Health.

## Usage Notes

This data set can serve as the basis for researchers to develop, evaluate, and optimise machine learning and deep learning models to predict treatment outcomes and support health professionals in the diagnosis, prognosis, treatment and control of tuberculosis. As a result, the burden on already overstretched health systems and economies, particularly those in disadvantaged regions around the world, can be reduced by accelerating the restoration. Furthermore, making data available enables researchers worldwide to carry out individual patient data meta-analysis and thereby generating more robust evidence on clinical and public health.

## Supplementary information

Supplementary Table 1

## Data Availability

The code used to pre-process the data set is publicly available on GitHub and is accessible through the link: https://github.com/dotlab-brazil/tuberculosis_preprocessing.
