# Supplementary material for: A Brazilian classified data set for prognosis of tuberculosis, between January 2001 and April 2020
Source: Sci Data. 2022 Dec 15;9:771. doi: 10.1038/s41597-022-01892-4 (PMC9753864; doi:10.1038/s41597-022-01892-4)
Supplement: Supplementary file 1 — Supplementary Table 1 [file 41597_2022_1892_MOESM1_ESM.docx]

| **Attributes names** | **Attributes descriptions** | **Total N=964,099** | **Cured N=927,909** | **Died N=36,190** |
| --- | --- | --- | --- | --- |
| CS_SEXO | Sex, % |  |  |  |
|  | Female | 337,343 (35.0) | 327,797 (35.3) | 9,546 (26.4) |
|  | Male | 626,587 (65.0) | 599,946 (64.7) | 26,641 (73.6) |
|  | Undefined | 169 (0.0) | 166 (0.0) | 3 (0.0) |
| CS_RACA | Race, (%) |  |  |  |
|  | White | 299,253 (31.0) | 289,015 (31.1) | 10,238 (28.3) |
|  | Black | 118,048 (12.2) | 113,093 (12.2) | 4,955 (13.7) |
|  | Yellow | 8,749 (0.9) | 8,482 (0.9) | 267 (0.7) |
|  | Admixed | 400,847 (41.6) | 383,934 (41.4) | 16,913 (46.7) |
|  | Indigenous | 11,471 (1.2) | 11,089 (1.2) | 382 (1.1) |
|  | Ignored | 125,731 (13.0) | 122,296 (13.2) | 3,435 (9.5) |
| TRATAMENTO | Patient entry situation,% |  |  |  |
|  | New case | 818,553 (84.9) | 792,407 (85.4) | 26,146 (72.2) |
|  | Relapse | 60,792 (6.3) | 57,950 (6.2) | 2,842 (7.9) |
|  | Re-entry after abandonment | 38,353 (4.0) | 35,222 (3.8) | 3,131 (8.7) |
|  | Don't know | 9,138 (0.9) | 8,444 (0.9) | 694 (1.9) |
|  | Transfer | 35,312 (3.7) | 33,783 (3.6) | 1,529 (4.2) |
|  | Post-death | 1,849 (0.2) | 1 (0.0) | 1,848 (5.1) |
|  | Ignored | 102 (0.0) | 102 (0.0) | - |
| RAIOX_TORA | Chest X-ray result, % |  |  |  |
|  | Suspicious | 745,157 (77.3) | 716,969 (77.3) | 28,188 (77.9) |
|  | Normal | 45,550 (4.7) | 44,411 (4.8) | 1,139 (3.1) |
|  | Other pathology | 9,582 (1.0) | 8,947 (1.0) | 635 (1.8) |
|  | No accomplished | 163,691 (17.0) | 157,466 (17.0) | 6,225 (17.2) |
|  | Ignored | 119 (0.0) | 116 (0.0) | 3 (0.0) |
| TESTE_TUBE | Tuberculin skin test result, % |  |  |  |
|  | No reactor | 36,835 (3.8) | 35,495 (3.8) | 1,340 (3.7) |
|  | Weak reactor | 15,301 (1.6) | 15,042 (1.6) | 259 (0.7) |
|  | Strong reactor | 89,605 (9.3) | 88,792 (9.6) | 813 (2.2) |
|  | Not performed | 821,948 (85.3) | 788,185 (84.9) | 33,763 (93.3) |
|  | Ignored | 410 (0.0) | 395 (0.0) | 15 (0.0) |
| FORMA | Clinical form of tuberculosis, % |  |  |  |
|  | Pulmonary | 815,686 (84.6) | 785,523 (84.7) | 30,163 (83.3) |
|  | Extrapulmonary | 123,293 (12.8) | 119,532 (12.9) | 3,761 (10.4) |
|  | Pulmonary+Extrapulmonary | 25,005 (2.6) | 22,749 (2.5) | 2,256 (6.2) |
|  | Ignored | 115 (0.0) | 105 (0.0) | 10 (0.0) |
| AGRAVAIDS | AIDS associated  with tuberculosis, % |  |  |  |
|  | Yes | 57,723 (6.0) | 52,571 (5.7) | 5,152 (14.2) |
|  | No | 518,380 (53.8) | 496,327 (53.5) | 22,053 (60.9) |
|  | Not performed | 1 (0.0) | 1 (0.0) | - |
|  | Ignored | 387,995 (40.2) | 379,010 (40.8) | 8,985 (24.8) |
| AGRAVALCOO | Alcohol consumption associated  with tuberculosis, % |  |  |  |
|  | Yes | 116,165 (12.0) | 106,752 (11.5) | 9,413 (26.0) |
|  | No | 523,144 (54.3) | 503,407 (54.3) | 19,737 (54.5) |
|  | Ignored | 324,790 (33.7) | 317,750 (34.2) | 7,040 (19.5) |
| AGRAVDIABE | Diabetes associated  with tuberculosis , % |  |  |  |
|  | Yes | 56,402 (5.9) | 52,906 (5.7) | 3,496 (9.7) |
|  | No | 568,204 (58.9) | 542,971 (58.5) | 25,233 (69.7) |
|  | Not performed | 1 (0.0) | 1 (0.0) | - |
|  | Ignored | 339,492 (35.2) | 332,031 (35.8) | 7,461 (20.6) |
| AGRAVDOENC | Mental disease associated  with tuberculosis, % |  |  |  |
|  | Yes | 18,134 (1.9) | 16,784 (1.8) | 1,350 (3.7) |
|  | No | 601,675 (62.4) | 574,754 (61.9) | 26,921 (74.4) |
|  | Ignored | 344,290 (35.7) | 336,371 (36.3) | 7,919 (21.9) |
| AGRAVOUTRA | Others diseases associated  with tuberculosis, % |  |  |  |
|  | Yes | 75,854 (7.9) | 69,183 (7.5) | 6,671 (18.4) |
|  | No | 386,942 (40.1) | 370,709 (40.0) | 16,233 (44.9) |
|  | Ignored | 501,303 (52.0) | 488,017 (52.6) | 13,286 (36.7) |
| BACILOSC_E | Sputum smear for  Acid-fast bacillus (AFB),  1st sample, % |  |  |  |
|  | Positive | 525,168 (54.5) | 508,871 (54.8) | 16,297 (45.0) |
|  | Negative | 211,415 (21.9) | 204,010 (22.0) | 7,405 (20.5) |
|  | In progress | 217,773 (22.6) | 205,942 (22.2) | 11,831 (32.7) |
|  | Not performed | 9,640 (1.0) | 8,983 (1.0) | 657 (1.8) |
|  | Ignored | 103 (0.0) | 103 (0.0) | - |
| BACILOS_E2 | Sputum smear for  Acid-Fast bacillus (AFB),  1st sample, % |  |  |  |
|  | Positive | 155,845 (16.2) | 150,749 (16.2) | 5,096 (14.1) |
|  | Negative | 82,110 (8.5) | 78,473 (8.5) | 3,637 (10.0) |
|  | Not performed | 133,793 (13.9) | 124,102 (13.4) | 9,691 (26.8) |
|  | Ignored | 592,351 (61.4) | 574,585 (61.9) | 17,766 (49.1) |
| BACILOSC_O | Smear other material for  Acid-Fast bacillus (AFB),  2nd sample, % |  |  |  |
|  | Positive | 19,827 (2.1) | 18,946 (2.0) | 881 (2.4) |
|  | Negative | 29,609 (3.1) | 28,584 (3.1) | 1,025 (2.8) |
|  | Not performed | 593,447 (61.6) | 575,231 (62.0) | 18,216 (50.3) |
|  | Ignored | 321,216 (33.3) | 305,148 (32.9) | 16,068 (44.4) |
| CULTURA_ES | Result of sputum culture  for M. tuberculosis, % |  |  |  |
|  | Positive | 121,293 (12.6) | 117,567 (12.7) | 3,726 (10.3) |
|  | Negative | 64,506 (6.7) | 62,841 (6.8) | 1,665 (4.6) |
|  | In progress | 42,627 (4.4) | 41,043 (4.4) | 1,584 (4.4) |
|  | Not performed | 733,646 (76.1) | 704,521 (75.9) | 29,125 (80.5) |
|  | Ignored | 2,027 (0.2) | 1,937 (0.2) | 90 (0.2) |
| HIV | Result of serology  for the acquired  immunodeficiency virus, % |  |  |  |
|  | Positive | 65,466 (6.8) | 59,961 (6.5) | 5,505 (15.2) |
|  | Negative | 478,373 (49.6) | 463,544 (50.0) | 14,829 (41.0) |
|  | In progress | 68,223 (7.1) | 66,953 (7.2) | 1,270 (3.5) |
|  | Not performed | 350,989 (36.4) | 336,535 (36.3) | 14,454 (39.9) |
|  | Ignored | 1,048 (0.1) | 916 (0.1) | 132 (0.4) |
| RIFAMPICIN | Rifampicin drugs, % |  |  |  |
|  | Yes | 639,766 (66.4) | 621,610 (67.0) | 18,156 (50.2) |
|  | No | 8,521 (0.9) | 6,826 (0.7) | 1,695 (4.7) |
|  | Ignored | 315,812 (32.8) | 299,473 (32.3) | 16,339 (45.1) |
| ISONIAZIDA | Isoniazid drugs, % |  |  |  |
|  | Yes | 639,620 (66.3) | 621,466 (67.0) | 18,154 (50.2) |
|  | No | 8,418 (0.9) | 6,729 (0.7) | 1,689 (4.7) |
|  | Ignored | 316,061 (32.8) | 299,714 (32.3) | 16,347 (45.2) |
| ETAMBUTOL | Etambutol drugs, % |  |  |  |
|  | Yes | 231,427 (24.0) | 220,935 (23.8) | 10,492 (29.0) |
|  | No | 395,454 (41.0) | 386,405 (41.6) | 9,049 (25.0) |
|  | Ignored | 337,218 (35.0) | 320,569 (34.5) | 16,649 (46.0) |
| ESTREPTOMI | Streptomi drugs, % |  |  |  |
|  | Yes | 7,770 (0.8) | 7,178 (0.8) | 592 (1.6) |
|  | No | 607,795 (63.0) | 589,249 (63.5) | 18,546 (51.2) |
|  | Ignored | 348,534 (36.2) | 331,482 (35.7) | 17,052 (47.1) |
| PIRAZINAMI | Pirazinami drugs, % |  |  |  |
|  | Yes | 633,834 (65.7) | 615,743 (66.4) | 18,091 (50.0) |
|  | No | 13,390 (1.4) | 11,648 (1.3) | 1,742 (4.8) |
|  | Ignored | 316,875 (32.9) | 300,518 (32.4) | 16,357 (45.2) |
| ETIONAMIDA | Ethionamide drugs, % |  |  |  |
|  | Yes | 8,966 (0.9) | 8,509 (0.9) | 457 (1.3) |
|  | No | 606,141 (62.9) | 587,492 (63.3) | 18,649 (51.5) |
|  | Ignored | 348,992 (36.2) | 331,908 (35.8) | 17,084 (47.2) |
| OUTRAS | Others drugs, % |  |  |  |
|  | Yes | 9,569 (1.0) | 8,786 (0.9) | 783 (2.2) |
|  | No | 566,324 (58.7) | 548,934 (59.2) | 17,390 (48.1) |
|  | Ignored | 388,206 (40.3) | 370,189 (39.9) | 18,017 (49.8) |
| TRAT_SUPER | Supervised treatment, % |  |  |  |
|  | Yes | 316,579 (32.8) | 307,470 (33.1) | 9,109 (25.2) |
|  | No | 339,043 (35.2) | 327,451 (35.3) | 11,592 (32.0) |
|  | Ignored | 308,477 (32.0) | 292,988 (31.6) | 15,489 (42.8) |
| DOENCA_TRA | Acquired the disease  as a result of the  working conditions/situation % |  |  |  |
|  | Positive | 12,780 (1.3) | 12,599 (1.4) | 181 (0.5) |
|  | Negative | 415,302 (43.1) | 401,882 (43.3) | 13,420 (37.1) |
|  | Not performed | 1 (0.0) | 1 (0.0) | - |
|  | Ignored | 536,016 (55.6) | 513,427 (55.3) | 22,589 (62.4) |
| BACILOSC_1 | Sputum smear for  Acid-Fast bacillus (AFB),  1st month, % |  |  |  |
|  | Positive | 156,038 (16.2) | 152,237 (16.4) | 3,801 (10.5) |
|  | Negative | 155,774 (16.2) | 154,016 (16.6) | 1,758 (4.9) |
|  | Not performed | 248,122 (25.7) | 236,735 (25.5) | 11,387 (31.5) |
|  | Not applicable | 33,200 (3.4) | 31,740 (3.4) | 1,460 (4.0) |
|  | Ignored | 370,965 (38.5) | 353,181 (38.1) | 17,784 (49.1) |
| BACILOSC_2 | Sputum smear for  Acid-Fast bacillus (AFB),  2nd month, % |  |  |  |
|  | Positive | 61,752 (6.4) | 61,040 (6.6) | 712 (2.0) |
|  | Negative | 319,550 (33.1) | 317,986 (34.3) | 1,564 (4.3) |
|  | Not performed | 368,968 (38.3) | 356,565 (38.4) | 12,403 (34.3) |
|  | Not applicable | 33,295 (3.5) | 31,808 (3.4) | 1,487 (4.1) |
|  | Ignored | 180,534 (18.7) | 160,510 (17.3) | 20,024 (55.3) |
| BACILOSC_3 | Sputum smear for  Acid-Fast bacillus (AFB),  3rd month, % |  |  |  |
|  | Positive | 13,293 (1.4) | 13,064 (1.4) | 229 (0.6) |
|  | Negative | 217,622 (22.6) | 216,727 (23.4) | 895 (2.5) |
|  | Not performed | 325,485 (33.8) | 313,558 (33.8) | 11,927 (33.0) |
|  | Not applicable | 33,354 (3.5) | 31,852 (3.4) | 1,02 (4.2) |
|  | Ignored | 374,345 (38.8) | 352,708 (38.0) | 21,637 (59.8) |
| BACILOSC_4 | Sputum smear for  Acid-Fast bacillus (AFB),  4th month, % |  |  |  |
|  | Positive | 10,955 (1.1) | 10,795 (1.2) | 160 (0.4) |
|  | Negative | 317,154 (32.9) | 316,461 (34.1) | 693 (1.9) |
|  | Not performed | 403,910 (41.9) | 392,082 (42.3) | 11,828 (32.7) |
|  | Not applicable | 33,391 (3.5) | 31,886 (3.4) | 1,505 (4.2) |
|  | Ignored | 198,689 (20.6) | 176,685 (19.0) | 22,004 (60.8) |
| BACILOSC_5 | Sputum smear for  Acid-Fast bacillus (AFB),  5th month, % |  |  |  |
|  | Positive | 3,683 (0.4) | 3,571 (0.4) | 112 (0.3) |
|  | Negative | 206,606 (21.4) | 206,206 (22.2) | 400 (1.1) |
|  | Not performed | 330,761 (34.3) | 319,365 (34.4) | 11,396 (31.5) |
|  | Not applicable | 33,380 (3.5) | 31,874 (3.4) | 1,506 (4.2) |
|  | Ignored | 389,669 (40.4) | 366,893 (39.5) | 22,776 (62.9) |
| BACILOSC_6 | Sputum smear for  Acid-Fast bacillus (AFB),  6th month, % |  |  |  |
|  | Positive | 4,311 (0.4) | 4,175 (0.4) | 136 (0.4) |
|  | Negative | 347,432 (36.0) | 347,036 (37.4) | 396 (1.1) |
|  | Not performed | 359,221 (37.3) | 347,747 (37.5) | 11,474 (31.7) |
|  | Not applicable | 33,383 (3.5) | 31,876 (3.4) | 1507 (4.2) |
|  | Ignored | 219,752 (22.8) | 197,075 (21.2) | 22,677 (62.7) |
| AGRAVDROGA | Other illicit drugs used by the patient   at the time of notification, % |  |  |  |
|  | Yes | 35,265 (3.7) | 33,361 (3.6) | 1904 (5.3) |
|  | No | 262,647 (27.2) | 251,348 (27.1) | 11,299 (31.2) |
|  | Ignored | 666,187 (69.1) | 643,200 (69.3) | 22,987 (63.5) |
| AGRAVTABAC | Tobacco consumption associated  with tuberculosis , % |  |  |  |
|  | Yes | 63,118 (6.5) | 58,936 (6.4) | 4,182 (11.6) |
|  | No | 236,287 (24.5) | 226,972 (24.5) | 9,315 (25.7) |
|  | Ignored | 664,694 (68.9) | 642,001 (69.2) | 22,693 (62.7) |
| DIAS_EM_TRATAMENTO | Number of days that the patient  was in treatment,  Mean rounded (SD) | 205 (103) | 211 (98) | 56 (130) |
| IDADE | Age, Mean rounded (SD) | 40 (17) | 39 (17) | 52 (18) |

**Supplementary Table 1.** General data set attributes description
